# Extramedullary Plasmacytoma of the Tonsil with Nodal Involvement

**DOI:** 10.1155/2010/302656

**Published:** 2010-07-20

**Authors:** Simon Bazaadut, Dilshard Soodin, Pradeep Singh, Alhossain Khalafallah, Shannon Withers, Scott Taylor, Ruchira Fernando

**Affiliations:** ^1^Department of Otolaryngology, Launceston General Hospital, Tasmania, Australia; ^2^Department of Oncology & Haematology, Launceston General Hospital, Tasmania, Australia; ^3^School of Human Life Sciences, University of Tasmania, Australia; ^4^Department of Pathology, Launceston General Hospital, Tasmania, Australia

## Abstract

We present a rare case of extramedullary plasmacytoma of the palatine tonsil with cervical lymph node involvement treated by surgical resection. A 58-year-old Caucasian male presented with a solitary 3 cm × 3 cm jugulodigastric lymph node and was found to have an ipsilateral tonsillar swelling. The involved tonsil and lymph node were surgically resected after two inconclusive fine-needle aspirates, and plasmacytoma was confirmed histologically and by immunocytochemistry. Adjuvant radiotherapy was not indicated as adequate resection was achieved at surgery. We also highlight the challenges of diagnosis when fine-needle aspiration is inconclusive and the need for careful planning before surgery.

## 1. Introduction

Extramedullary plasmacytomas are rare tumours which usually occur in the upper aerodigestive tract; it is even much rarer for them to originate from the tonsil. It is important to exclude systemic myeloma or multilesional disease in these patients. Long-term monitoring is mandatory to detect early local recurrence or conversion to multiple myeloma.

## 2. Case Presentation

A 58-year-old Caucasian male was referred to the otolaryngology outpatient clinic from the oncology department with a complaint of a right-upper neck mass which had been growing for one year. The mass was painless and had rapidly increased in size during the preceding 3 months. The patient reported no loss of weight and had no other masses. He denied swallowing or breathing difficulties. There was no history of exposure to tuberculosis. He was a farmer and life-long nonsmoker. On physical examination, he was a well-nourished man with a palpable, freely mobile nontender right level II lymph node measuring 3 cm × 3 cm. Oropharyngeal examination showed a moderately enlarged right tonsil. The rest of the physical examination was unremarkable. Rigid direct laryngoscopy showed a normal laryngopharynx and hypopharynx.

**Figure 1:**
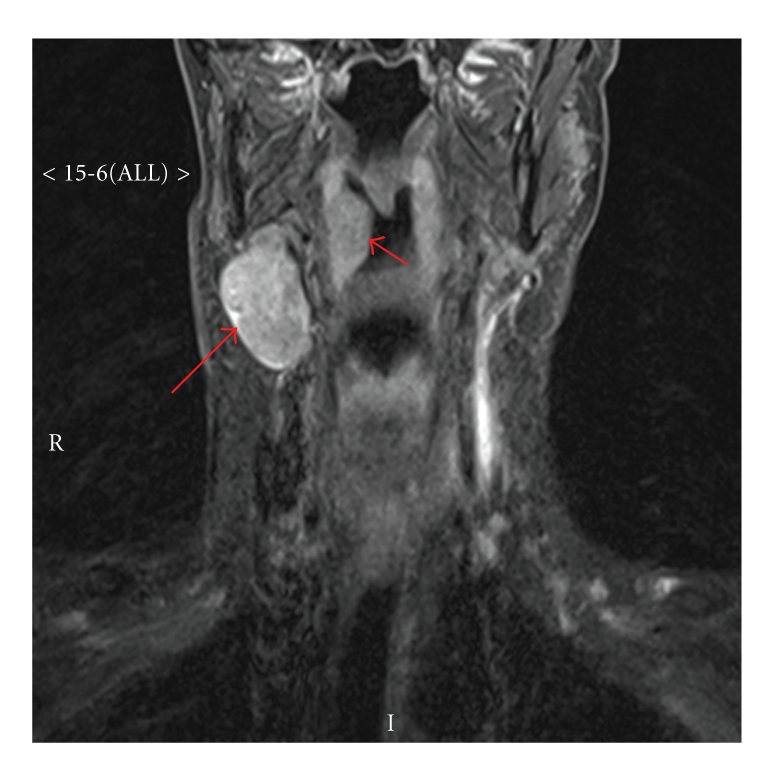
MRI of the neck showing an enlarged lymph node (long arrow) and a bulge in the right tonsillar fossa (short arrow) suspicious for primary tumour.

Due to a possible vascular relation to the mass, an MRI of the neck was done, showing a sharply defined ovoid 3 × 3 × 4.5 cm nonspecific soft tissue mass with heterogeneous enhancement, likely a lymph node, with a mass-like bulge in the right tonsillar fossa suspicious for a primary tumour (Figure 1). This supported the clinical diagnosis of a tonsillar primary lesion with a regional nodal deposit.

Two previous fine-needle aspirates (FNAs) of the neck mass had already been undertaken by the oncologists, but these were inconclusive. The aspirates were heavily blood stained and showed small tissue fragments composed of small lymphoid cells admixed with histiocytes. The sparse material suggested an inflammatory/reactive process, and flow cytometry was nondiagnostic. Therefore an excision biopsy with frozen sections was recommended for definitive diagnosis. If this revealed a squamous cell carcinoma, the operation would proceed to a selective neck dissection. The patient consented to the procedure and underwent a right tonsillectomy and excision of the enlarged lymph node. Frozen sections suggested a plasmacytoma and therefore a neck dissection was not carried out.

**Figure 2:**
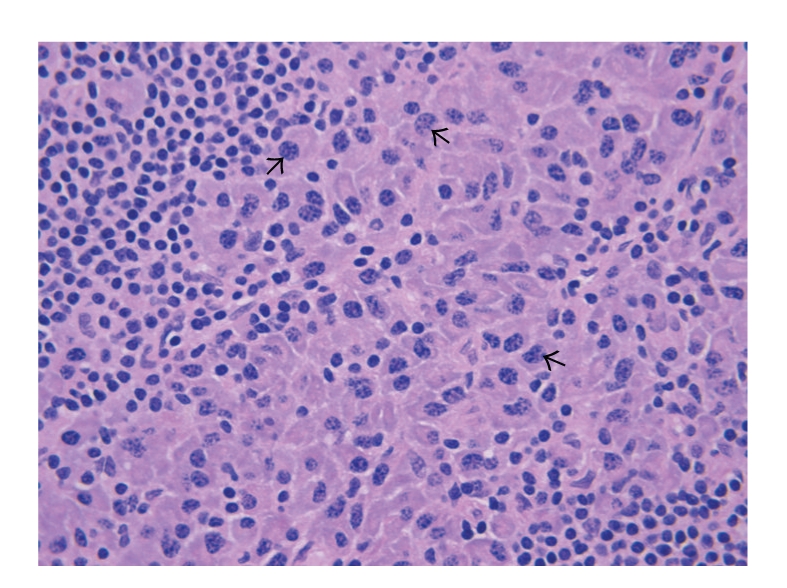
Lymph node with diffuse infiltrate of plasma cells including atypical binucleated and multinucleated forms (black arrows) (H & E stain, x40).

**Figure 3:**
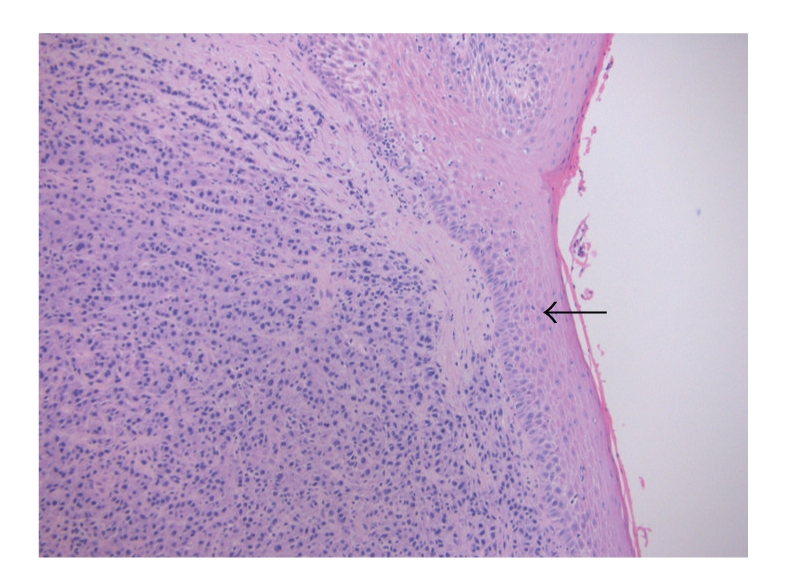
Tonsillar tissue with intact surface squamous epithelium (arrow) and underlying diffuse infiltrate of neoplastic plasma cells (H & E stain, x10).

Histologies of both the tonsillar mass and the lymph node were similar, showing effacement of normal architecture and extensive infiltration with diffuse sheets of neoplastic cells possessing plasmacytoid morphology with eccentric nuclei exhibiting “clock-faced” nuclear chromatin pattern that typically represent plasma cells. Binucleate and multinucleate forms were also present (Figure 2). The tonsillar lesion was fairly circumscribed and the epithelium was not infiltrated (Figure 3).

**Figure 4:**
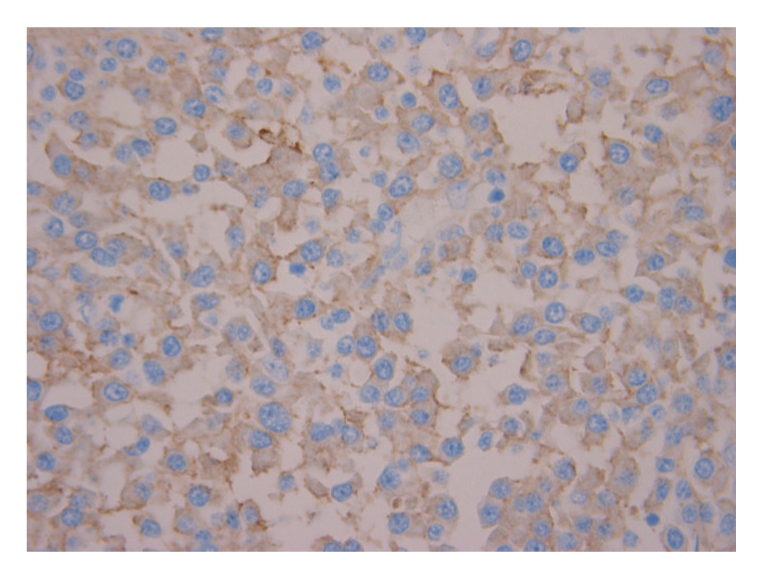
CD138 immunostain was positive, confirming plasma cells (x40).

Further immunohistochemical analysis on the specimens showed CD138 plasma cell marker positivity (Figure 4), while CD20 and CD3 were negative, with kappa light chain restriction. This profile was in keeping with plasmacytoma. Serum protein electrophoresis showed the presence of monoclonal IgG kappa with normal levels of residual immunoglobulins. Skeletal survey, bone marrow biopsy, serum-free light chain, and urine analysis for Bence-Jones protein showed normal results. 

Six months after surgery the patient remained well. Repeat immunoglobulin assay at the 3-month postoperative review showed that the monoclonal IgG had returned to normal levels, with normal light chains and no evidence of multiple myeloma on bone marrow biopsy and no lytic lesions on skeletal survey. Repeat investigations at the 6-month mark remained negative.

## 3. Discussion

Plasmacytomas are malignant proliferations of plasma cells that occur either in bone (medullary) or in soft tissue (extramedullary). Medullary or extramedullary disease may present as either solitary or multiple lesions. The systemic disease involving multiple lesions in bones (called multiple myeloma) is the commonest plasma cell dyscrasia, accounting for over 90% of all plasma cell malignancies. 

The exact incidence of extramedullary plasmacytomas (EMP) is unknown, but they are rare, accounting for only 3%-4% of all plasma cell malignancies [1, 2]. It is estimated that 80%–90% of EMP develop in the head and neck region [2–4]. There are no identifiable risk factors. In an extensive retrospective study of reported cases between 1905 and 1997, Alexiou et al. [2] found that 82.2% of cases of EMP occurred in the upper aerodigestive (UAD) tract, with 10.5% involving the tonsil or soft palate. They also found that the most common areas of the UAD affected were the nasal cavity or paranasal sinuses (43%), nasopharynx (18.3%), and oropharynx (17.8%). Nodal involvement of EMP in the UAD was reported in only 7.6% of cases [2].

EMP of the tonsil usually presents as a unilateral tonsillar swelling, but at least one case of bilateral involvement of the palatine tonsil has been reported [5]. Evaluation of the patient begins with a full history and clinical examination, including oral examination and nasopharyngoscopy.

FNA may provide inconclusive results as it is sometimes difficult to distinguish inflammatory or reactive conditions from evolving tumours. However, FNA may be useful in SCC or recurrent EMP, especially if used in conjunction with a core biopsy and subsequent immunocytochemistry [6, 7]. 

Imaging studies such as computer tomographic (CT) scanning, magnetic resonance imaging (MRI), and positron emission tomographic (PET) scanning usually provide useful information on the extent of local disease. Anatomical localization is enhanced with integrated PET/CT scanning, especially when localising EMPs outside the head region where it is proven to be more sensitive than MRI [8]. 

Preferably all patients should be referred to a hematologist for further workup to exclude multiple myeloma or disseminated disease. Baseline full blood examination, serum calcium, urea, and electrolytes should be performed in addition to serum protein electrophoresis and immunofixation, urine Bence-Jones proteins, skeletal survey, and bone marrow examination. The abnormal plasma cells of EMPs originate from one cell line and all produce the same immunoglobulin, usually an IgG kappa light chain. In some patients this can be detected as a small monoclonal peak on immunoelectrophoresis called the M spike. With successful eradication of disease, the M spike disappears and this can be demonstrated on subsequent followup. 

Solitary EMP is managed with radiotherapy, surgery, or both. If a complete surgical resection is achieved, then no adjuvant radiotherapy is required as in our case [8]. However, regardless of whichever option is used, regular monitoring is warranted as the risks of local recurrence and development of multiple myeloma still exist and can occur many years after treatment of the initial tumour. In retrospective, nonrandomised studies, local recurrence was observed in 22% of cases [2]. 

We recommend reviews at 3 monthly intervals for the first year, 6 monthly for another year, and yearly thereafter for life. At each visit, in addition to a history and physical examination, laboratory testing including complete blood count, urea, creatinine and electrolytes, immunoelectrophoresis, and free light chains should be performed. This will be in addition to yearly monitoring with MRI (which was our initial imaging modality). At any hint of recurrence such as new M spike, a full reevaluation is required including bone marrow biopsy and an integrated CT/PET.
